# ESR2 regulates cytokinin dynamics for plant regeneration

**DOI:** 10.1093/plphys/kiaf230

**Published:** 2025-06-05

**Authors:** Syed Adeel Zafar, Jitesh Kumar

**Affiliations:** Center for Plant Cell Biology, Department of Botany and Plant Sciences, University of California, Riverside, CA 92507, USA; Assistant Features Editor, Plant Physiology, American Society of Plant Biologists; Department of Plant and Microbial Biology, University of Minnesota, Saint Paul, MN 55108, USA; Center for Precision Plant Genomics, University of Minnesota, Saint Paul, MN 55108, USA

Plant regeneration, defined as the ability to develop new organs or even an entire plant from a single cell or an explant, is a fundamental biological process governed by phytohormones. Cytokinins (CKs) and auxin are central to directing the cellular and developmental pathways necessary for regeneration. CKs regulate a wide range of developmental processes, including cell division, shoot and root meristem activity, vascular differentiation, and organogenesis. The biosynthesis of CKs is primarily carried out by ISOPENTENYLTRANSFERASE (IPT) enzymes, which catalyze the initial and rate-limiting step in CK biosynthesis. CK signals are initially perceived by the histidine kinase receptors (AHKs), which undergo auto-phosphorylation upon CK binding. Signals are then transduced by authentic histidine phosphotransfer proteins (AHPs) and finally relayed to type-A and type-B response regulators. The sophisticated CK signaling cascade enables spatiotemporal control of developmental decisions, often through interaction with auxin (Augstein and Carlsbecker 2018; Kieber and Schaller 2018).

Auxin and CKs usually have antagonistic effects in controlling developmental fate in plants. For example, in *Arabidopsis* root meristem development, Indole-3-acetic acid promotes root meristem elongation while CK inhibits meristem growth (de Vries et al. 2016). Thus, a tight regulation of CK and auxin biosynthesis and signaling is critical for normal plant development. CKs play a crucial role in shoot regeneration from explant and callus tissue, making them a key component of plant tissue culture. CKs are used in tissue culture together with auxins, and the ratio of CK to auxin in the growth medium determines the fate of callus tissue. A high CK to auxin ratio promotes green callus and shoot regeneration, while a low ratio promotes root formation. Thus, understanding how biosynthesis and sensitivity of CKs are regulated at the molecular level is critical for improving shoot regeneration efficiency, particularly in recalcitrant species or genotypes less amenable to regeneration. Research over the past few decades has significantly enhanced our understanding of the regulators of CK biosynthesis and signaling, as well as their roles in callus and shoot regeneration. Notably, the AP2/ERF transcription factors ENHANCER OF SHOOT REGENERATION 1 (ESR1) and ESR2 have been identified as key players of callus formation and shoot regeneration in *Arabidopsis* via CK-dependent and independent pathways (Banno et al. 2001; Ikeda et al. 2006). ESR2, in particular, has been shown to induce shoot regeneration through transcriptional regulation of CUP-SHAPED COTYLEDON 1 (CUC1), and *ESR2* overexpression enabled CK-independent shoot regeneration (Ikeda et al. 2006). Despite these insights, the downstream targets through which ESR2 mediates CK biosynthesis or signaling in the context of green callus formation remain unknown.

In the latest issue of *Plant Physiology*, Durán-Medina et al. (2025) filled this knowledge gap by demonstrating that ESR2 modulates CK homeostasis by regulating the expression of *IPT5* and *AHP6*, key components of CKs biosynthesis and signaling, respectively. To investigate these effects, the authors used *β-*estradiol–inducible *ESR2 Arabidopsis* line *35S::ESR2:ER* (*ESR2:ER*), which allows controlled activation of *ESR2* expression. ESR2 induction in the seedlings led to arrested leaf and root growth, and extended periods of induction resulted in the formation of green calli along the entire root length (Figure A and B). Given the known role of CKs in green callus formation, the authors measured and discovered a significant increase in endogenous CK levels in ESR2-induced plants, suggesting that ESR2 regulates CK levels.

**Figure.**
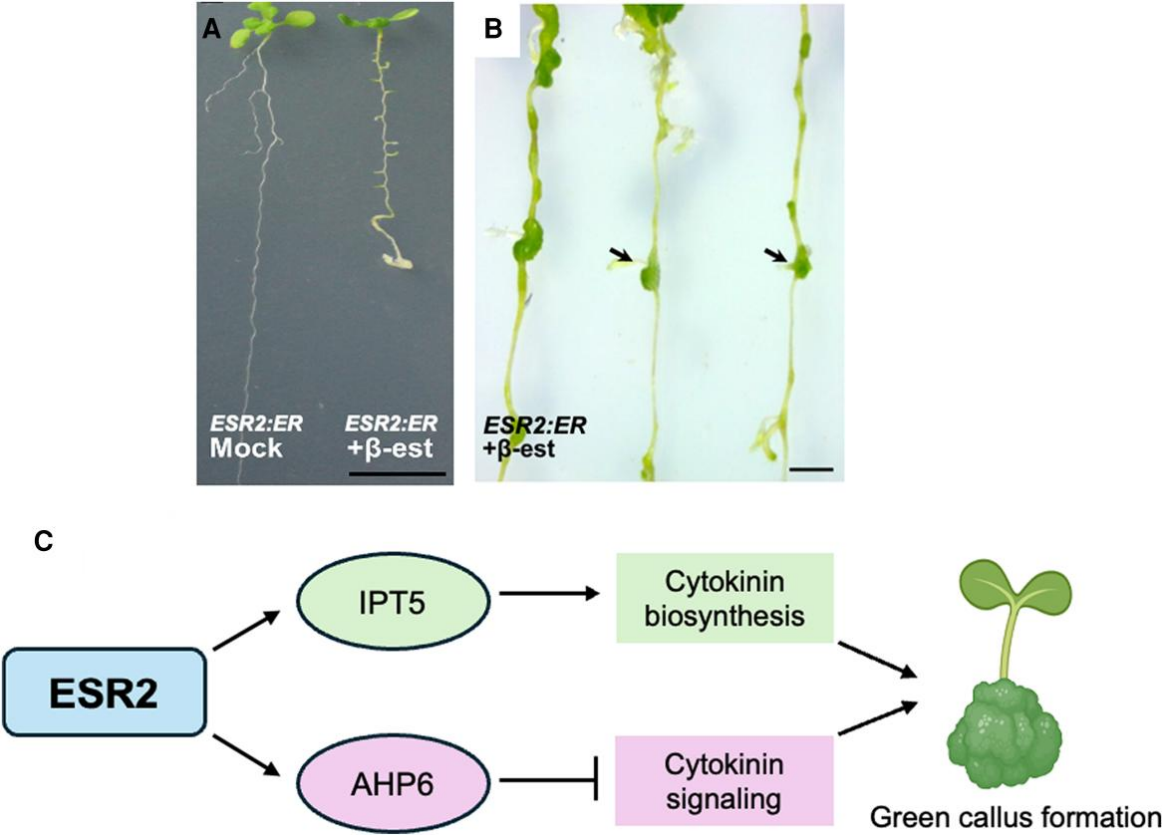
ESR2 regulates the expression of genes involved in CK pathways and green callus formation. **A)** A comparison between roots of mock-treated and *β*-estradiol–induced *ESR2:ER Arabidopsis* seedlings. **B)** Calli developing from the tissue near or at the base of young lateral roots after 21 days of ESR2 induction. **C)** Hypothetical model showing ESR2 mediated regulation of IPT5 and AHP6 for regulating CK biosynthesis and signaling, respectively. These hormonal changes promote green callus formation. Images (A and B) are adopted from Durán-Medina et al. (2025).

An increase in endogenous CK levels intrigued authors to investigate the roles of ESR2 in regulating the genes involved in the CK pathway. They used reverse transcription quantitative PCR and GUS/GFP reporter assays with a focus on *IPT5*, encoding the rate-limiting enzyme in the CK biosynthesis pathway, and *AHP6*, a suppressor of CK signaling. Reverse transcription quantitative PCR revealed elevated expression of both genes within 30 min of ESR2 induction. Furthermore, *ESR2:ER* line harboring *IPT5::GUS* and *AHP6::GFP* constructs revealed enhanced expression in the aerial organs and root vasculature. Notably, *ESR2:ER AHP6::GFP* line displayed increased protoxylem vessels, suggesting their potential role in vascular development.

To test the role of IPT5 and AHP6 in green callus formation and vascular patterning, the authors created the *ESR2:ER* inducible line in the *ipt5* and *ahp6* mutant background. While *ESR2:ER* induction caused stunted leaf and root development, this phenotype was lost in the *ESR2:ER ipt5* and *ESR2:ER ahp6* plants. Similarly, roots of the *ESR2:ER* induced line have shown green calli formation along the entire root length, but this green calli formation was severely reduced and absent in *ESR2:ER ipt5* and *ESR2:ER ahp6* plants, respectively. Notably, exogenous CK (BAP) application has restored visible calli in the *ESR2:ER ipt5* and, to a lesser extent, in *ESR2:ER ahp6* induced plants, suggesting a direct role of IPT5 and AHP6 in green calli formation. Interestingly, *ESR2:ER ipt5* roots exhibited a reduced number of protoxylem vessels compared to the *ESR2:ER* induced plants, while *ESR2:ER ahp6* plants showed a disorganized pattern of root vasculature. The results suggest the opposing roles of *IPT5* and *AHP6* in vascular development directed by ESR2.

Finally, yeast 1-hybrid and transient luciferase reporter assays demonstrated that ESR2 directly binds to the regulatory elements (such as transcription factor binding sites) in the promoter regions of *IPT5* and *AHP6*, validating them as direct downstream targets of ESR2 in the gene regulatory network. Together, these results reveal that ESR2 modulates both CK biosynthesis and signaling in a tissue-specific manner by directly regulating *IPT5* and *AHP6* (Figure C). However, the mechanisms underlying the distinct tissue-specific expression patterns of *IPT5* and *AHP6* remain unclear. One possibility is that ESR2 may require different partners to bind to different promoters or that variations in cis-regulatory elements regulate tissue-specific expression (Zafar and Bailey-Serres 2025).

In conclusion, Durán-Medina et al. (2025) provide compelling evidence that *ESR2* controls callus shoot regeneration and vascular development by regulating CK biosynthesis and signaling via targeting *IPT5* and *AHP6* (Figure C). These findings advance our understanding of the *ESR2*-mediated transcriptional regulatory networks underlying shoot regeneration. However, it remains unclear what the upstream regulators of *ESR2* are and how *ESR2* integrates with auxin-mediated pathways, since loss-of-function mutants of *ESR2* tomato ortholog *LEAFLESS* (*LFS*) fail to produce cotyledons and leaves by maintaining a local auxin maxima at the site of lateral primordia initiation (Capua and Eshed 2017). It also remains to be determined whether *ESR2* function is conserved across the species and can be engineered to optimize regeneration potential across plant taxa. Future studies can be directed toward identifying upstream regulators of *ESR2*, elucidating its crosstalk with auxin pathways, and evaluating its functional conservation across diverse plant lineages.

## Data Availability

No new data were generated or analysed in support of this research.

## References

[kiaf230-B1] Augstein F, Carlsbecker A. Getting to the roots: a developmental genetic view of root anatomy and function from Arabidopsis to lycophytes. Front Plant Sci. 2018:9:1410. 10.3389/fpls.2018.01410 30319672 PMC6167918

[kiaf230-B2] Banno H, Ikeda Y, Niu QW, Chua NH. Overexpression of Arabidopsis ESR1 induces initiation of shoot regeneration. Plant Cell. 2001:13(12):2609–2618. 10.1105/tpc.010234 11752375 PMC139476

[kiaf230-B3] Capua Y, Eshed Y. Coordination of auxin-triggered leaf initiation by tomato *LEAFLESS*. Proc Natl Acad Sci U S A. 2017:114(12):3246–3251. 10.1073/pnas.1617146114 28270611 PMC5373412

[kiaf230-B4] de Vries J, Fischer AM, Roettger M, Rommel S, Schluepmann H, Bräutigam A, Carlsbecker A, Gould SB. Cytokinin-induced promotion of root meristem size in the fern Azolla supports a shoot-like origin of euphyllophyte roots. New Phytol. 2016:209(2):705–720. 10.1111/nph.13630 26358624 PMC5049668

[kiaf230-B5] Durán-Medina Y, Díaz-Ramírez D, Herrera-Ubaldo H, Di Marzo M, Cruz-Valderrama JE, Guerrero-Largo H, Ruiz-Cortés BE, Gómez-Felipe A, Reyes-Olalde JI, Colombo L, et al ESR2 orchestrates cytokinin dynamics leading to developmental reprogramming and green callus formation. Plant Physiol. 2025:198(1):kiaf182. 10.1093/plphys/kiaf182 40343942

[kiaf230-B6] Ikeda Y, Banno H, Niu QW, Howell SH, Chua NH. The ENHANCER OF SHOOT REGENERATION 2 gene in Arabidopsis regulates CUP-SHAPED COTYLEDON 1 at the transcriptional level and controls Cotyledon development. Plant Cell Physiol. 2006:47(11):1443–1456. 10.1093/pcp/pcl023 17056621

[kiaf230-B7] Kieber JJ, Schaller GE. Cytokinin signaling in plant development. Development. 2018:145(4):dev149344. 10.1242/dev.149344 29487105

[kiaf230-B8] Zafar SA, Bailey-Serres J. Decoding the evolution of C4 photosynthesis. Trends Plant Sci. 2025; 10.1016/j.tplants.2025.02.008 40038015

